# Life Themes and Interpersonal Motivational Systems in the Narrative Self-construction

**DOI:** 10.3389/fpsyg.2017.01897

**Published:** 2017-10-27

**Authors:** Fabio Veglia, Giulia Di Fini

**Affiliations:** Department of Psychology, University of Turin, Turin, Italy

**Keywords:** narration, life stories, narrative themes, interpersonal motivational systems, meaning-making, theoretical model

## Abstract

What makes unique and unrepeatable individuals is their ability to write their own story attributing meaning, sharing it through narration, giving coherence to the information related to the interpersonal motivational systems, and creating alternative hierarchies to those biologically provided by the genetic code. Through clinical narratives and narrative literature, we can observe the recurrence of specific topics, across time and different cultures. Hence, we wondered whether there are some evolutionary suggestions that guide us in the construction of the narrative-autobiographical contents. In this article we proposed a theoretical-clinical hypothesis about the existence of a biological disposition to identify as fundamental six Life Themes (LTs) that contribute to defining the image of the self and the world: Love, Personal Value, Power, Justice, Truth, and Freedom. Besides the innumerable narratives dependent upon context, there may be many ways of telling stories that, instead, would be reported to these few essential themes. A narrative review of the literature about these concepts follows the systematic explanation of the perspective about the LTs as attractors of meaning. The manuscript considers also the process of co-construction of meanings within the interpersonal relationships and the influences of these on the narratives. In particular, we focused on the importance of episodic and autobiographical memory related to the attachment and significant figures, in the construction of the personal story and the LTs. We also explained the possible clinical implications of the theoretical hypothesis of LTs. Within clinical conversations, the LTs could be expressed rigidly or, otherwise, in a confused way. The lack of narrative integration may lead to the dominance of chaos or rigidity that generates suffering. A better comprehension of the LTs in patients’ narrations could be useful to identify a narrative profile about the areas of greatest suffering related to the idea of self and the world, as well as to construct an adequate care plan.

## Functions of Narration and the Search for Meaning

A complex innate perceptive and motor network drives human behavior from the earliest moments of life (Lambruschi et al., 2004) to generate a series of representations that regulate, select and process perceptual information and social actions (Liotti, 1996). These representations are adaptive suggestions called interpersonal motivational systems (IMSs) (Liotti and Gilbert, 2011). Caregiving, attachment as wells as competitive/ranking, sexual-mating, and co-operative systems are specific for each critical situation that can be addressed through an appropriate interpersonal position and are activated and deactivated based on whether their goal is met (Liotti and Monticelli, 2008). In the history of human development, alongside the more archaic “reptilian” motivations (related to the regulation of basic homeostatic systems, survival and reproduction) and to the “limbic” social motivations (MacLean, 1984; Liotti, 1994), there are motivations associated with the neocortical brain (related to the intersubjective meaning system) which allow the organism to carry out an evolutionary passage (Veglia, 1999).

Liotti and Monticelli (2008) identified the most recent epistemic motivational systems (EMSs) supported by the activity of neocortical circuits and neural networks that mediate interhemispheric connectivity. These systems involve the ability to share and co-construct personal meanings and mental states.

**FIGURE 1:**
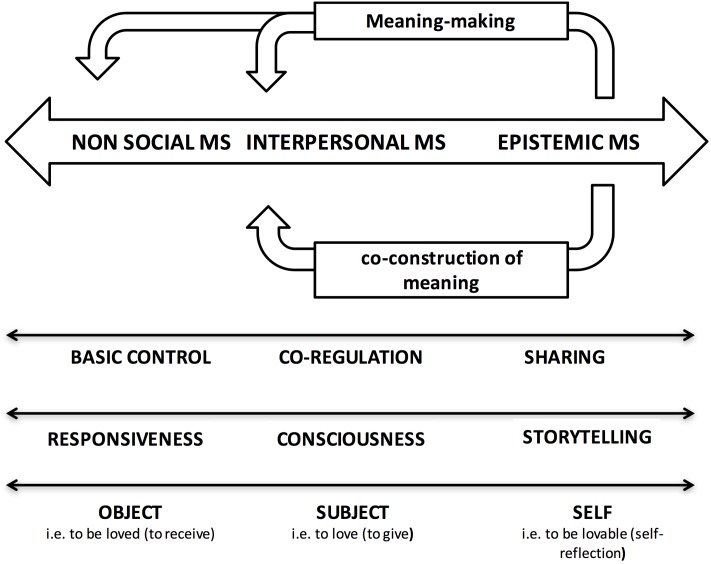
The scheme summarizes the evolutionary steps according to the Triune Brain theory (MacLean, 1984). The development of epistemic motivational systems allows man to attribute and co-construct meanings to the world and the activity of the older motivational systems. On the bottom the figure illustrates the different trajectories that the thematic narrative can follow in the constant attempt to interpret the flow of incoming information. The bi-directionality of the arrows indicates the individual’s ability to move in both directions along the continuum that connects the three evolutionary steps.

The new evolutionary goal directs an individual to attribute meaning to his/her life by giving order, consistency and unity to the knowledge possessed and incarnated through the activation of the oldest limbic and non-social motivational systems to harmoniously organize his/her vision of self, others and the world. This evolutionary innovation, integrated with the purposes and advantages of previous goals, introduces the need to link elements of reality, perceived through logical and causal links, and to ask questions about the world (**Figure 1**). The individual makes meanings and creates new ways of conceiving and inventing reality (Bruner, 1997). In addition, in interpreting human action in terms of intentional states, he/she tends to tell a story that assigns a plausible meaning to the self and others (Feldman, 1997). Thus, the tools provided to carry out this new evolutionary mandate include the sense of time, integration of semantic and episodic memories, construction of mental images, languages, and metacognitive functions. The sense of time and the ability to link events distant from one another in space and time has been suggested to be closely related to the ability to tell stories and, in turn, to the development of communication based on the language (Ferretti, 2016).

The narration hypotheses, supported by Bruner (1990, 2003), have been demonstrated by neurobiological studies based on neuroimaging (Young and Saver, 2001; Mar, 2004, 2011; Spreng and Mar, 2012). Young and Saver (2001) identified four types of “dysnarrativia” (narrative impairments following focal lesions in different regions of the neural network that mediates the creation of narrative). These types of autobiographic experiences include “arrested narratives,” “unbounded narratives,” “under narration,” “denarration,” and involve the networks responsible for the encoding of autobiographical memories, language, and organization of information in narrative frames (the amygdalo-hippocampal system, the left peri-Sylvian region, and the frontal cortices and their subcortical connections). These defects in narrative construction destabilize the individual’s personality. The story comprehension has been found connected to a network of frontal, temporal and cingulate areas that are related to working-memory and theory-of-mind processes (Mar, 2004).

Other authors suggested that the roots of narrative are closely related to human sensorimotor intelligence; thus, meaning-making would be embodied and linked to the organization of the purposeful movement (Delafield-Butt and Trevarthen, 2015).

## Thematic Content of Narratives

What makes individuals unique and unrepeatable is their ability to write their own story by attributing meaning to external and internal reality, giving order and cohesion to information and knowledge related to the work of interpersonal motivational systems, and building alternative hierarchies to biological goals provided by the genetic code (Veglia, 1999). The opportunity to make stories contributes to creating that space of self-determination for which biological constraints do not constitute inviolable limits, but resources to draw what is possible. The peculiarity of a personal story also involves the specificity of the development of some narrative themes that are shaped like the expression of neocortical motivational systems. Along the gradual and progressive definition of the image of the self and the world in historical and narrative form (Damasio, 1999, 2010), the individual confronts himself/herself with the particular thematic constraints, which act as organizers of meaning in the experience and manifest themselves across cultures and organizations of personality (Guidano, 1987). These themes are constraints, but also opportunities, for the development of the narrative plot as they guide the attribution of meaning to experience, leaving creative freedom to choose from countless variations (Veglia, 1999, 2013).

McAdams (1996, 2001) identified some “nuclear” and constant themes that permeate life stories. He distinguished two main critical themes around which individuals extend the accounts of their existence: agency and communion. The agency is based on self-identification and affirmation. Concepts such as strength, power, mastery, autonomy, separation and independence are included. Life stories are organized around a central nucleus that sees the individual protagonist and master of his/her life. Inside agency it is distinguished as: *self-mastery; status/victory*, namely the recognition of a prestigious position within interpersonal relationships; *achievement*/*responsibility*, related to the realization, achievement of goals and assumption of responsibility; *empowerment*, which is growth and self-strengthening.

Communion, on the other hand, is indicative of the processes of sharing within interpersonal relationships. It is linked to experiences of affiliation, union and intimacy. Communion includes *love/friendship*, or erotic love or friendship with another person; *dialog*, which concerns forms of mutual and non-instrumental communication; *caring/help*, which concerns care, assistance, physical, material, social or emotional support; *unity/togetherness*, which refers to the feeling of union, harmony, intimacy, synchrony, loyalty, closeness, and solidarity with a group of people, community, or any other type of aggregation of individuals.

Within constructionism, Ugazio (2013) identified some *semantic polarities* (i.e., meaning dimensions and the antagonists among them) around which each family organizes their conversations. Such “meaning organizers” identify what is relevant to a group, and articulates their experiences. Four sets of semantic polarities have been identified, nourished by specific emotions, called the *semantic of freedom, goodness, power, and belonging*. What is particularly salient is the reciprocal placements that the subject and meaningful people take in the family dialog with respect to the critical theme (Castiglioni et al., 2013).

## The Theoretical–Clinical Hypothesis: Cross-Cultural Life Themes (LTs)

The existence of a biological disposition to identify as fundamental some LTs within which it is possible to carry out all our main biological goals in a structured and coherent manner has been hypothesized (Veglia, 1999). When IMS (Lichtenberg, 1989; Lichtenberg et al., 1992; Liotti, 2001) work, they follow in interdependence an evolutionary stage that involves the building of a coherent vision of the self and the world through which men/women can think and tell in a wide space of time (Guidano, 1987): LTs arise at this level. Reptilian and limbic motivations are lived by people as the primary needs to satisfy. Neocortical motivations are felt not only as a necessity but as something that allows us to approach what we consider the “true” purpose: the ultimate goal of life. In fact, neocortical motivations could be understood as a genetic invitation to carry out some LTs (Veglia, 1999). They are created on the basis of the significant life events, of individuals’ interpretation, of the consequences they have derived. They contribute to defining the image of the self and the world.

Life themes can be considered as frameworks of common meaning to the human species and transversal to different cultures. Bruner (1990) described the “narrative genres” which impose thematic constraints on how to tell themselves and the world, but also allow for creative and personal variations. Narrative themes, therefore, should be considered not like scripts, but the primary, irreducible, and generative scenarios of countless, unique and unrepeatable texts narrated by individuals. While imposing thematic limits, the development of the neocortex offered the individual the freedom to create infinite variations on the subject that are as flexible as they allow the dialog between the different parts of the self.

Veglia (1999), in the first formulation of his theoretical hypothesis, tried to identify the primary thematic nuclei. Such systematization, in accordance with McAdams (1996, 2001) studies, included two thematic areas: *control/power* and *semantics/sharing.* The two thematic areas, through the formation of Me/You (sharing) and I/It (control) (Buber, 1923) relationships, contribute to the development of their own story and the achievement of increasingly complex goals. The area of *control/power* concerns issues that involve the possibility of having control over the survival, mating and maintenance of the wellbeing for self and offspring through automatic and partially intentional actions. The themes of the *semantic/sharing* thematic area underline the importance of interpersonal relationships and the influence they have on the attribution of meaning. These areas allow us to develop more themes and form the plot of our knowledge and our story. Each theme has intrinsic and optional development lines, each of which, through their continuous interconnections, influence in different ways the perception of self, behaviors, future design, cognitive styles, relational styles, articulation of knowledge, as well as the construction of organized social systems, cultures, and stories.

In a second formulation of the theoretical hypothesis, Veglia (2013) identified six recurring, transcultural, and trans-organizing LTs, within the previous two thematic areas, that would be expressed as the attractors of meaning and would be delineated through phylogenetic evolution and ontogenetic development of the various relational positions, and interpersonal motivational systems, such as attachment, caregiving, competitive/rank, sexual-mating, cooperation and belonging.

**FIGURE 2:**
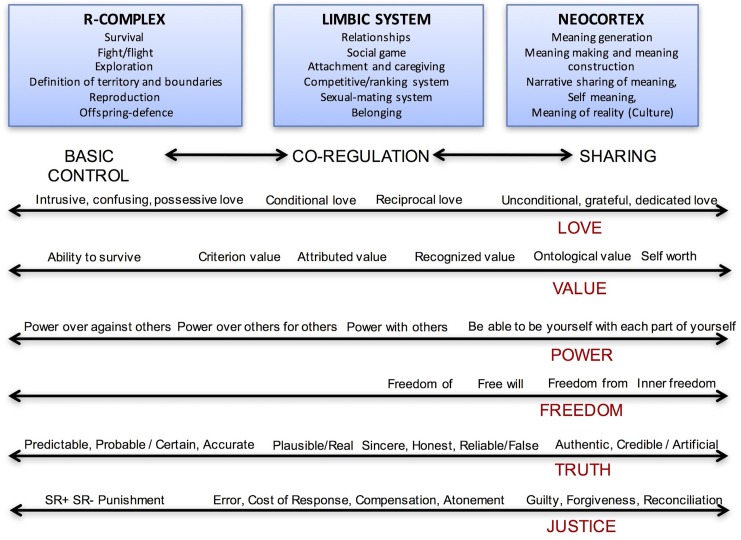
Life themes and their different meanings along the continuum of evolution. The six themes meanings along the two-way arrows represent the tools that allow the narration to move through the three evolutionary steps.

Narrative themes have been identified with reference to the analysis of mythology, narrative literature, anthropological, philosophical, psychological, and neuroscientific essays as well as the analysis of hundreds of clinical stories (see **Figure 2**).

The analysis, carried out over several years by a research group within the Department of Psychology of the University of Turin and a clinical group within the Crocetta Clinical Center in Turin, both led by Fabio Veglia, developed by applying focus-group and consensus-conference methods. After a broad brainstorming, constraints were created for generating conceptual maps using five main criteria.

The first criterion was the theme’s irreducibility (i.e., the inability to consider the narrative content as part of another theme, regardless of the possibility to combine several themes to generate stories). The second criterion was the utmost independence from contextual characteristics (i.e., the inability to bring about the existence of the narrative content under consideration to a peculiar feature of a single, historical, geographical, or anthropological context by its nature not being necessarily recurring or non-generalizable). The third criterion was the presence of the theme in every culture and every historical period examined, regardless of the innumerable modes and possible recombination of its development. The fourth criterion was the intrinsic structural capacity of the theme (also called the “semantic attractor” or “narrative theme” or “life theme”) to attract and generate meaning both in the form of expressions (even non-linguistic) and in the construction of the self and the dialog between parts of the self. The final criterion was not meeting the inclusion criterion of the simple high occurrence of arguments, contents, mechanisms, processes, mental states, attributes, meaning organizations, life events, mental representations that are often referred to as “themes.”

The six Life Themes that emerged have been named: Love, Personal Value, Power, Freedom, Truth and Justice.

Subsequently, the six themes were subjected to qualitative analysis using the transcripts of the Adult Attachment Interview (AAI, George et al., 1985, unpublished). This strategy was in accordance with the hypothesis of continuity between the expression of the limbic/relational attachment/caregiving system and the neocortical system related to the attribution of meaning to the self and the world through the memory of significant interactions. This study highlighted the presence of the LTs in the AAI transcripts even though they were not the subject of the questions. Furthermore, these LTs were linked to the different states of mind with respect to the attachment. It allowed dissolving reservations on the theme of *Justice* which, according to the first hypothesis, seemed to be a derivative of the narrative of other themes, not respecting the criterion of irreducibility (Di Fini, unpublished doctoral dissertation).

An exploratory study of psychological literature was conducted on the six attractors of meaning identified by the theoretical–clinical hypothesis of Veglia (1999, 2013). With regard to the nationality of the authors of identified articles, most of the studies were conducted by Americans, followed by Canadians, Israelis, Dutch, English, Germans, and Australians. With respect to the historical trend of scientific articles from 1950 to the present day, frequency analysis showed a strong growth since the 1970s. This upward trend seems to be steady until today.

Referring to the content of the articles, there was great heterogeneity in the areas in which each theme was investigated: from the area of general psychology, to the discipline of psychotherapy, to social and developmental psychology.

The narrative review of the literature about each construct follows the systematic explanation of the perspective about the LTs as attractors of meaning. Instead, for the description of the axes, along which LTs may position themselves, see the section “The theoretical–clinical hypothesis: cross-cultural axes.”

### Love

In terms of evolution, the Love theme is in direct continuity of the experience of caring (caregiving), the search for proximity (attachment), sexual intimacy (sexual-mating system), friendship (co-operation) and is supported by neocortical motivations that allow men/women to be conscious and free.

The analysis of the substance of the love experience is beyond the boundaries of this work and of this discipline. This object of study has been always immense, mysterious, and irreducible for everyone who has approached it. However, the need to live and tell love by different expressions, interests, moves and guides all human beings.

Love, so different in its forms, but unique in something of its essence, is more than the proximity and protection by a safe base. Love is *philia*, *agape*, *caritas*, *eros*, friendship, shared and incarnated attribution of meanings through the body, its signs, emotions, and also (but not necessarily) through the word (Veglia, 1999). In this article, we refer to all these ways of love and its countless other possible manifestations.

We can hypothesize three narrative positions used to describe love: to be loved, or the object of someone’s love, regardless of the quality of that love; to be loving, as a subject of love, though not necessarily in a relationship based on reciprocity; to be lovable, as perception, representation, and self-narrative.

Obviously in terms of presence/absence there may be those who have not been loved, disliked, and not recognized as a lovable person.

The positive/negative quality of the narration involves the position of having been badly loved, or having expressed his/her own love toward others in the form of control and possession, or having obsessively doubted even in the absence of well-founded motives of his/her own amiability.

The most complete expression of love narrative is based on the human capacity to generate and share the meaning of new tales and the high integrative abilities of the neocortex. It involves unconditional love, able to recognize others, be devoted to others, and be respectful of their boundaries, inexhaustible, manifest, and proactive. Hence, potentially, this is the love of parents, friends, and, in some cases, of partners.

The experiences of love based on emotional co-regulation are more conditioned, but still beneficial. These put an “I love you if you love me” constraint by reducing mutual freedom, but also the serious risks of suffering related to unconditional love.

**FIGURE 3:**
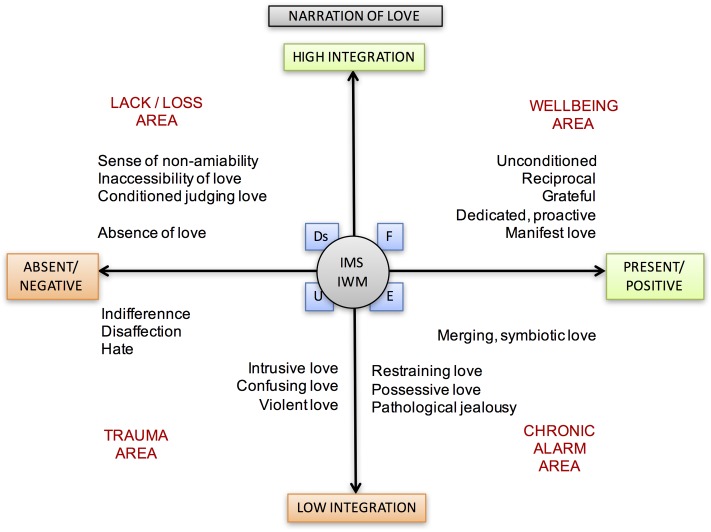
Narration of Love theme and its different meanings according to its position in the areas between axes.

Conversely, in its less integrated and more controllable expressions, love can be a semantically negative experience. A person who invades, uses, constrains, limits, confuses, scares, threatens, or humiliates the object of his/her love turns narrative into a toxic and traumatic experience for each person, but ultimately for himself/herself. Many wounds of the body and spirit are suffered in the name of love. The graphical representation of this Theme is reported in **Figure 3**.

In literature *Love* seems to be considered among the most intense feelings of human beings and among the most investigated ones. In fact, most of the articles considered for this topic tried to explain what love is by offering a wide variety of models and approaches.

Maslow (1962) made a distinction between Deficiency love (D-love), which emerges from the insecurity and lesser emotional need of an individual, and Being love (B-love), which emerges from a high emotional need of an individual. Livingston (1980) considered love to be a process of reducing uncertainty. The definition proposed by Pope (1980) seemed to proffer a great deal of agreement and seemed to capture the many affections and cognitions associated with love.

Rubin (1970, 1973) developed a “Love Scale” based on three theoretical components: the “Affiliative and dependent need,” the “Predisposition to help,” and the “Exclusiveness and absorption.” A scale of love measurement based on a different theory was proposed by Steffen et al. (1984). It is based on Tennov’s (1979) theory of limerence, which defines it in terms of an intrusive cognitive activity, acute desire, addiction, and physical sensations in response to a loved one, painting it as an extreme romantic attachment to another person. Lee (1973) built a typology of styles of loving by analyzing the literature. This typology distinguishes six types of love (three primary and three secondary): *Eros* (romantic and passionate love); *Ludus* (love as interpersonal play between different partners); *Storge* (an inclination to fusion and friendship, without passion); *Mania* (a dependent and possessive love); *Pragma* (a rational calculation with focuses on the desirable attributes of the partner); *Agape* (a disinterested and unselfish love). Secondary styles are conceived as chemical compounds of the primary elements, and although all styles are interrelated, each one has qualitative independent properties.

Fehr (1988) identified a set of 68 characteristics of love, such as “love,” “in love,” and “liking,” which seem to have a clear prototypic structure in the sense that some features are good examples of the concept. In psychotherapy, Natterson (2003) observed that love is a fundamentally dynamic element of the therapeutic process as any intimate relationship. Our article portrays therapy as a process of mutual love subject to the many vicissitudes to which love is concerned.

### Value

The neocortex appears to push into estimating personal value and to consider it as a property of the self, hoping to see it recognized by others and thus shared. This theme is in continuity of the immediate evaluation of survival and reproduction capacity (R-complex) and the subsequent evaluation attributed by our conspecifics to our strengths, abilities, and competencies through the typical activation of interpersonal motivational systems (limbic system).

It involves types of value (considered to be a system of personal features that qualify an individual) that differ from each other according to the ways of assignment and attribution within interpersonal relationships. We distinguish, on the one hand, a *criterion* value if its attribution is based on external and quantifiable criteria and, on the other hand, an *ontological* value if its recognition describes the person’s mode of being, according to internal parameters. The first one leads to a permanence problem because it will be attributed to the person until the criteria are respected. The second one will not be exhausted because it will not be contingent or tied to specific conditions, but will represent the essence of the individual, which persists even after a total disregard of all his/her properties.

**FIGURE 4:**
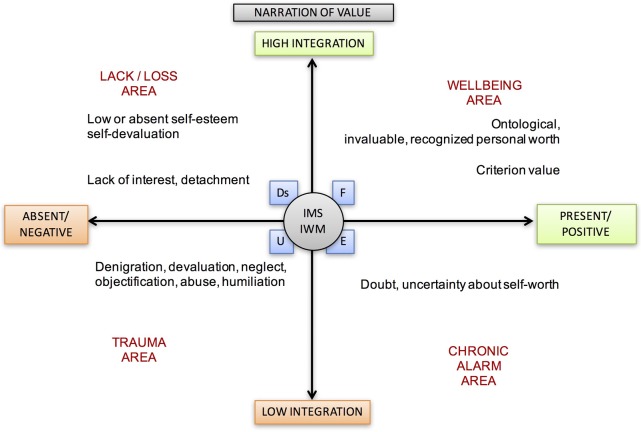
Narration of Value theme and its different meanings according to its position in the areas between axes.

As shown in **Figure 4**, in some cases, the concept of value assumes a relational connotation: we must seek dialog and relationship with others to continue to confirm and retain the feeling of having a value. Within interpersonal relationships, the value can be attributed, and generates for the individual a condition of expectation and dependence on the assignment and judgment imposed externally. On the contrary, it can be recognized in a climate of sharing and co-construction of the meaning. For a signal of personal value to be recognized as effective requires a negotiation and sharing of meanings. The estimating indicators do not necessarily have to coincide with the rank indicators produced by the expression of the competitive/ranking system (Veglia, 1999).

With respect to *Value*, it seems that scientific research focuses on self-esteem as an operationalization of the Value theme. In particular, this construct is present in the literature in the dual meaning of self-esteem and self-worth. The concept of self-esteem means self-evaluation by the subject if the criteria for estimation are externally decided; self-worth means self-evaluation by the subject if the criteria are internal. Developmental psychologists (Erikson, 1963; Sroufe, 1978) gave great emphasis to the role of the early emotional experiences in determining the sense of emotional wellbeing, personal value and self-esteem. Children, presumably, learn whether their environment is loving and satisfying or hostile and frustrating, before the development of a complex cognitive system capable of evaluating specific beliefs about the self.

Drawing on the theory of self-determination, Pyszczynski et al. (2003) argued that people seem to feel better if their self-esteem is based on abstract and unique features rather than if it is based on more superficial aspects. However, according to Crocker et al. (2003), self-esteem is partly based on receiving the approval and liking of others (Mead, 1934; Coopersmith, 1967). In fact, self-esteem is generally correlated with the positivity of what people think others think of them (Coopersmith, 1967; Bowlby, 1982).

Furthermore, religious belief is also moderately associated with self-esteem and other aspects of psychological wellbeing (Baker and Gorusch, 1984; Bergin et al., 1987; Nelson, 1989). Religion can have positive effects on self-esteem through the belief that a person is loved, esteemed and unique in God’s eyes. Another potential contingency for self-esteem is the morality or virtue of a person (Coopersmith, 1967; Benson and Lyons, 1991), because adherence to a moral code can lead to judgment that a person is good, moral and worthy.

### Power

**FIGURE 5:**
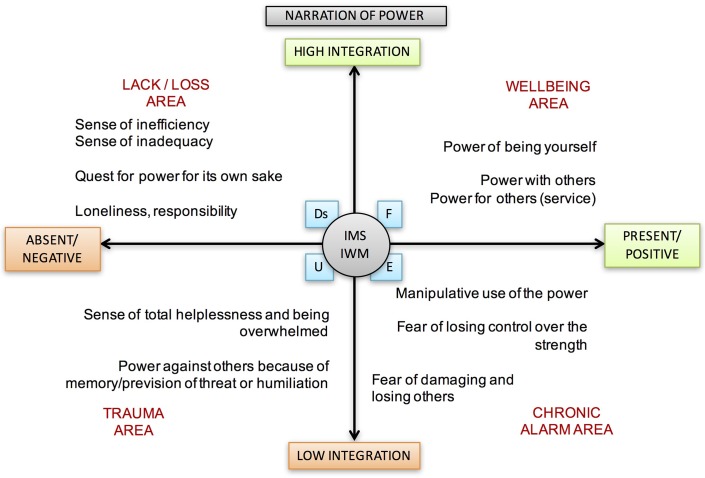
Narration of Power theme and its different meanings according to its position in the areas between axes.

At the limbic motivational level, the competitive/ranking system (Liotti, 2001) regulates the ability to compete with others to defend privileges and gain new benefits by defining social ranks and resource-regulated access. At the neocortical level, the Power theme is a narrative framework within which there is thinking and telling of the stories of competitions. It also refers to the personal sense of control of oneself, others, and events, accompanied by the individual’s awareness of being an active author of a project. Humans are obliged, for survival, to keep control of the physical and social environment to make more effective interactions with other conspecifics. Through metacognition, individuals make understandable to others the relationships and forms of control. The position of each of us with respect to control and being controlled contributes to the construction of the self and defines the perception of personal power (see **Figure 5**).

Power, because it not always involves shared possibilities, can include multiple effects within interpersonal relationships. *Power over others* is often synonymous with abuse and submission, and rarely is managed for the benefit of the group; it can become risky if its management represents a source of value. The *power for others* is exercised in favor of a single person or a group (power is a service). *Power with others* involves solidarity and co-operation among people who share it in collaboration for a common goal.

The most meaningful power is, ultimately, the ability of being freely and authentically yourself with every part of the self. It involves the ability to become accomplished without having to adapt to the desires of others. However, “in order to be me” it is necessary for the existence of a grateful and respectful “you.” This particular sense of power is similar to the concept of mastery (Semerari et al., 2003): management of individual mental states also in relation to the elaborated theory of the mental states of others.

In the literature, the *Power* theme seems to play a key part. The most frequently encountered perceptions for this Theme are “Power of” and “Power on,” which is otherwise called control.

Decades of research in sociology and psychology have shown that the perception of control is a strong predictor of physical and mental wellbeing (Rodin, 1986; Bandura, 1989; Fiske and Taylor, 1991; Thompson and Spacapan, 1991; Lachman and Burack, 1993). According to Wong (1992), life is an unstoppable search for control: much of our time is spent in the effort to reach the sense of control. Conversely, aggression and conflict, submission and domination, negotiation, and cooperation are just some of the ways people try to solve control issues. Another interesting development in this area was the discovery that people have beliefs about how they can control their behaviors, emotions and attitudes in the sense of modifying or regulating them (Baltes and Baltes, 1990; Brandtstadter et al., 1993; Skinner and Wellborn, 1994). In this case, beliefs about control contain perceptions of the degree to which individuals can produce the desired results or prevent unwanted ones within themselves. The need for expertise is closely related to other control constructs; nevertheless, the notion of a psychological need of effectiveness as a source of motivation is distinct from subjective, objective, and experiential control. The need for competence is often confused with the need for autonomy or self-determination and, therefore, the constructions of perceived control are often confused with the belief system resulting from autonomy experiences, such as the locus of causal attribution (Rodin, 1990). Hegarty (2007) reported that psychologists seem to have largely ignored the way power works in real communities: who exercises it, where it derives, and how it is used to influence the attitudes and behaviors of community members. Considerable attention has been paid to these important issues by sociologists and political scientists.

In their revision of the literature on power, Keltner et al. (2003) suggested that power activates a general tendency to approach, whereas lack of power activates a general tendency to inhibition. If this were true, power-loving individuals should exhibit greater orientation to action than those without power, regardless of the social consequences of their actions. However, other researchers showed that power not always leads to antisocial consequences, but may be the catalyst for achieving prosocial results that might not be realized otherwise. In addition, those studies demonstrated that power can be conceived not only as an aspect of social structure, but also as a cognitive structure that can be triggered by appropriate environmental stimuli.

Galinsky et al. (2003, 2006) defined power as the ability to control one’s own or others’ resources without social interference (Thibaut and Kelley, 1959; Keltner et al., 2003). In many conceptualizations of power, the ability to influence and control the behavior of others is of primary importance (French et al., 1959; Manz and Gioia, 1983; Oshiaki Imai, 1993; Copeland, 1994). This type of power has been called “social power” because it derives from an individual’s relationships with others (Fiske, 1993; Overbeck and Park, 2001). For Winter (1973), “power motive” designates the need to be authoritative, effective and have an impact on the surrounding world. Submitting to power is also a strong motivation to increase personal status or prestige. The Power theme in the sense of human possibility, and self-realization as the full affirmation of the most intimate essence of the person was taken up by McClelland (1975) in describing two tendencies of the individual: achievement and power motives. The first one is a type of personal achievement that uses feedback mechanisms to control one’s own success; the second one includes a form of decline in personal power, or in the shade of power over others, or in institutional (or even social) power.

In a clinical setting, De Varis (1994) stated that power in the therapeutic relationship is like a double-edged sword which can either release the patient from the chains of psychopathology or, unknowingly, reduce the patient’s empowerment.

The process of effective therapy provides considerable power and respect to the patient; it activates the patient’s ability to cure himself/herself and his/her active participation in the change process. This strengthens the patient, reduces his/her arbitrary sense of authority, and strengthens mutuality; it also increases, clarifies and reveals the patient’s personal power to himself/herself. In this process, the therapist, who was initially idealized, is finally recognized for his/her humanity, fallacy, and internal conflicts.

### Freedom

This theme refers to the possibility of free choice, which probably belongs only to humans. Freedom emerges with partial evolutionary discontinuity and along with the new narrative possibilities that bring us into our cognitive horizons and greatly increases the risks associated with choices and decisions. Hence, it also takes shape in our stories in relation to the limits and boundaries necessary to stifle the experiences of anguish, confusion and disorientation that would result from its tendency to infinity (Castiglioni et al., 2014).

The share of freedom in an individual’s life is indispensable for dealing with ethical issues and should be in balance with that portion of protective constraints that enables us to outline action plans in our life story. The limit of every personal freedom is the freedom of all other persons, so we must limit ourselves to receive mutual assurances. The notion of responsibility as the ability to guarantee safety within social relationships derives from this narrative theme. The Freedom theme is, simultaneously, the content and framework of the other themes, as well as the requirement for the existence of ontological value, of being yourself and of unconditional love. The freedom to act, express, and speak represents the different forms freedom can assume in the world of an individual’s meanings and to make sense, especially within meaningful relationships.

Apart from the freedom to express, choose and decide, we can conquer, with the process of individual growth, the partial freedom from our inner constraints, which we normally perceive as irreplaceable and which dramatically limit needs. Adults who take care of us, accompany us during childhood and adolescence, must support us in the struggle for liberation from the irrepressible impulse to immediately satisfy our needs. The inner discipline resulting from it can give us new wide spaces of semantic and procedural freedom.

**FIGURE 6:**
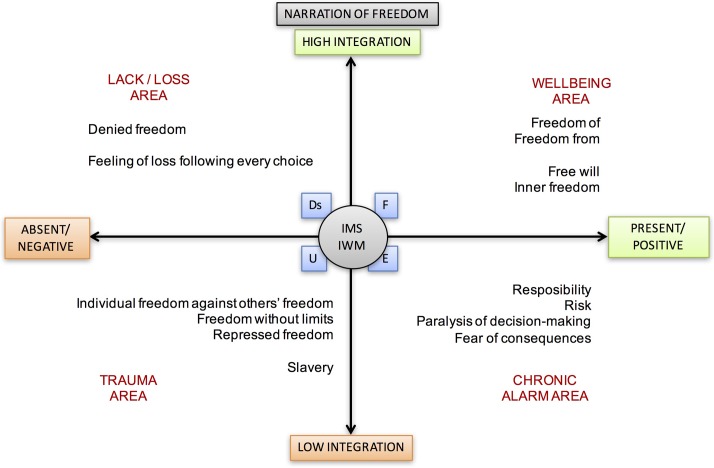
Narration of Freedom theme and its different meanings according to its position in the areas between axes.

Finally, we distinguish the concept of free will, in which the general possibility of choosing anything, regardless of its ethical value, and the idea of freedom as an opportunity to choose from the infinite opportunities to preach the “good” (Saint Augustine, 5th century AD). The problem in this case is not to opt for socially negative behaviors, but to assess whether this option is self-narrative without triggering internal conflicts. In other words, the condition for freedom is represented by the possibility of telling one’s actions, as well as guaranteeing access to the sharing and negotiation of their meaning. The increase in the degree of freedom and, simultaneously, of the flexibility with which it attributes meaning to experience, is the minimum requirement for any possibility of change. The graphical representation of this Theme is reported in **Figure 6**.

The basic problem pursued by psychologists who deal with *Freedom* comes from observing a broad belief in individual freedom and the indeterminacy of human action. It is important to recognize the distinction between freedom as a formal assumption of a psychological theory and personal conviction (Mandler and Kessen, 1974; Mandler, 1994).

Steiner (1970) introduced the concept of “perceived freedom.” His conceptual analysis distinguished between two aspects of perceived freedom: “Outcome Freedom” and “Decision Freedom.” The first is the estimated likelihood that the goal of each alternative course of action can be reached. The second is the degree to which the actor is seen as the critical determinant of the action, and not constrained by external conditions such as differences in the costs or benefits associated with the different rates of action. Once a value has been established for freedom of decision, Outcome Freedom influences the value of alternatives and changes the value of Decision Freedom.

In a 1997 publication, O’Connor et al. (1997) said that it is usually the negative aspect of freedom that leads people to approach psychotherapy: the desire to be rid of the weight of thoughts and emotions that reduce their freedom and act according to their wishes. In the course of psychotherapy, the positive aspect of freedom is cultivated in a context conducive to autonomy and self-regulation as desirable goals. However, the transition from the notion of “self-liberation” to that of “self-control” implies the exploration of the complex interaction between the subjective sensations of freedom and the objective limits placed upon it. People can become anxious and fearful in the face of what they perceive as too much freedom in the absence of significant boundaries for choices and behavior (Fromm, 1942).

### Truth

In terms of evolution, the topic of truth is linked to the need for living organisms to predict events as exact or probable in relation to the factual world. Unknowingly, the most archaic part of our central nervous system learns from experience, with reasonable chances of exercising sufficient control over the context despite the risk of developing superstitious behaviors.

Then, with the emergence of conscious awareness and intentionality, following the problem of prediction, there is the need to recognize and distinguish the true from false, or from deception and relational traps, or to pretend to gain relational benefits.

In its most recent semantic developments, however, this theme embraces and supports others as it directly represents the fulfillment of the third evolutionary mandate for the search for meaning. Here, truth is understood as the foundation for building meaning. The investigation of the truth within narrative plots means referring to its own way of telling the experience and explaining the continuous flow of time (Guidano, 1987).

**FIGURE 7:**
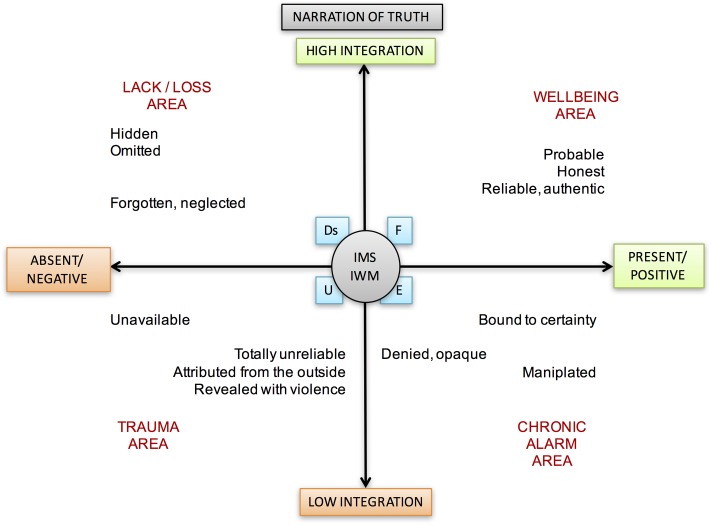
Narration of Truth theme and its different meanings according to its position in the areas between axes.

However, if a person, in contact with his/her narrative truth, fails to accept it and coherently integrate it into his/her own story, he/she can create a sort of self-deception, “accommodation and adjustment of the truth,” that makes it more compatible with the rest of the plot storytelling. It follows that there may be internal dissonances between the will to genuinely deepen meanings and the disturbance that derives from their examination. The Truth theme (see **Figure 7**) is carried out in full agreement with the constructivist view of knowledge as an incessant building-up based on experience, reflective reflection of one’s own experience, on personal experimentation, and not on the search for an absolute truth that is alien to the subject’s point of observation (Armezzani, 2004). It involves the search for the meaning created through the immediate comprehension of self-in-world, in which the other represents the necessary condition for validating and confirming our own perspective about the experience.

### Justice

The Justice theme is extremely complex in its semantics, narrative forms, historical and cultural differences, and even more in precarious attempts for good administration. For a long time, we were hesitant about its inclusion among LTs.

In fact, it is closely related to Value, still in tune with merciful Love (severe punishment or forgiveness?), and employed constantly in trying to moderate the narration and arbitrary exercise of Power, Freedom, and Truth. It could, therefore, be traced back to the narratives of the other five themes and depend on them in their semantics without respecting the criterion of irreducibility. In fact, the research that we have conducted on AAI transcripts would show an autonomous structure of this narrative theme.

The most critical and perhaps more archaic positions in the narrative of Justice are related to the indifference to injustice, inability to assume responsibility for the damage or suffering caused to others, lack of guilt and to disposition toward repair and reconciliation. The poor integration of the semantics related to justice, the strong push to have control over others with every means at the expense of others and the experience of serious and protracted suffered injustices expose individuals and communities to serious psychopathological risks.

**FIGURE 8:**
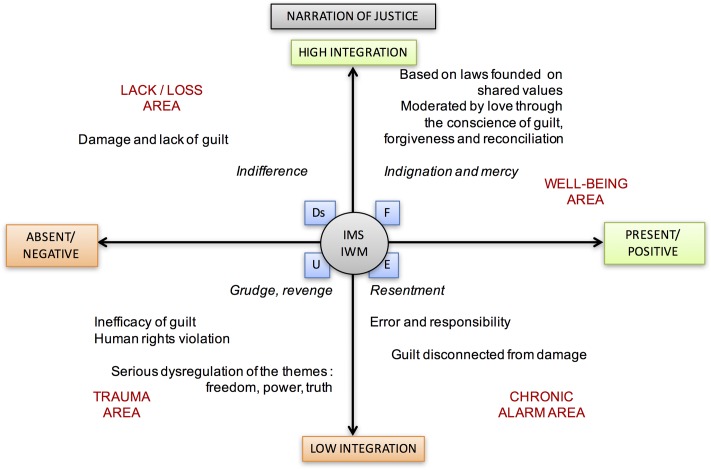
Narration of Justice theme and its different meanings according to its position in the areas between axes.

Conversely, questioning the foundations of justice, the commitment to build it through a system of laws based on shared values, as well as combating injustice by mutual recognition of the same rights and duties protect us from semantic relational trauma and participate in the construction of a cohesive self-rooted in belonging to one’s own community (see **Figure 8**).

At the evolutionary level, the narrative of the need for justice and the protest for injustice appears during childhood. However, its application is, for a long time, regulated by the outside and often, during adolescence, is perceived as a form of violence in a sometimes dramatic way of framing.

According to Colquitt (2001) *Justice* is involved in the field of organizational sciences. Justice is considered a social construction: consequently, an action is considered right if most individuals perceive it to be right. In particular, organizational justice can be described by focusing on the antecedents and consequences of two types of subjective perceptions: the correctness of the results of the distribution processes and the correctness of the procedures used to determine such distribution results. These forms of justice are typically referred to as “distributive justice” (Adams, 1965; Deutsch, 1975; Leventhal, 1976) and “procedural justice” (Thibaut and Walker, 1975; Leventhal et al., 1980). Efforts to understand the impact of justice on effective organizational functioning are included in the category of research on organizational justice (Greenberg, 1990).

Dzuka and Dalbert (2007) claimed that workplace justice matters to employees because these experiences meet the fundamental need to believe in a just world; on the contrary, experience of injustice can threaten this need by providing evidence of its infallibility (Cubela Adoric and Kvartuc, 2007). There are also empirical and theoretical reasons for considering belief in a fair world as a relatively stable conviction but, as Cubela Adoric and Kvartuc suggested, it is also conceivable that under certain conditions it may be weakened. A strong belief that the world is right will be linked to better mental health and wellbeing due to its association with the knowledge that helps us to function in everyday life, such as the confidence that we will be treated fairly by others and the perception of the significance of life events. According to many theorists of social justice, evaluations of fairness or iniquity are closely related to peoples’ merits (Lerner and Whitehead, 1980; Lerner, 1987; Major, 1994; Feather, 1999). Moreover, these attributes strongly preach the impressions of justice and congruent emotions such as anger and rancor. There is, thus, the possibility of defining justice explicitly and largely in terms of what is deserved. According to Ellard and Skarlicki (2002), the factors affecting the merits of victims and observers and, therefore, judgments on equity, may differ.

In summary, the large number of articles confirmed the importance of LTs as powerful motifs of meaning from which individual and social narratives unfold. These Life Themes, in fact, are also present in a wide range of disciplines.

The first evidence emerging from qualitative analyses is that psychological research tends to fragment themes (as they are considered according to this hypothesis) in multiple dimensions and constructs. Moreover, because of this fragmentation, it is not always easy to attribute a construct to one topic or another.

Faced with the vastness of the results in many fields of interest in psychology, there are few studies on LTs in the purely narrative context. Thus, the “limbic” motivations are often confused with the higher “neocortical” motivations related to the attribution of meaning. Instead, the theoretical–clinical hypothesis of Veglia (1999, 2013) refers to conceiving these narrative themes as powerful organizational predispositions of narratives.

It might be interesting to repeat investigation of the literature at a deeper level of complexity and specificity to detail more clearly how these attractors of meaning are referred to.

## The Theoretical–Clinical Hypothesis: Cross-Cultural Axes

**FIGURE 9:**
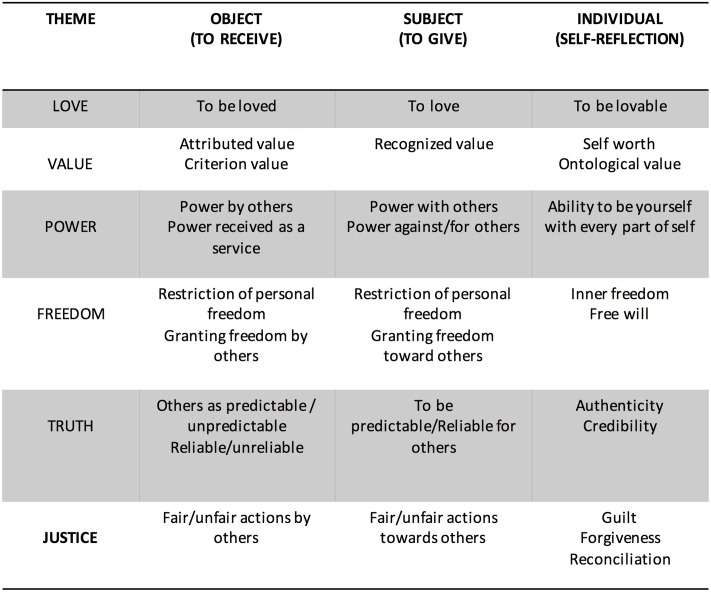
Meanings of the themes according to the interpersonal positions and movements.

The narrative, conversational and relational nature of the LTs makes it necessary to locate the narrator’s position and the direction of the “movement” associated with attributing meaning. Each person may alternatively be the object of attributing meaning to someone else, and the subject who attributes it to others and the world. Simultaneously, the individual can be the subject and the object of this action when he/she reflects on himself/herself. In **Figure 9**, we observe the six narrative themes studied in the three different positions and in the relational movements generated by them.

Study of the LTs has been formalized using three polarized axes. Each of these polarized axes describes a continuum of possible positions: starting from their common intersection at point 0, these axes identify areas of wellbeing and narrative suffering with all possible intermediate positions. Each of the areas is placed in connection with the four states of mind related to the AAI (*Free, Dismissing, Entangled*, and *Unresolved*).

At point 0, which generates the polarized directions of the three axes, early attachment/caregiving experiences and the related states of mind are represented. The three axes are described below.

### Basic Control vs. Co-regulation vs. Sharing of the Theme

The prevalence and alternation of top–down and bottom–up activation related to the different life stories and to the flow of energy and information (Siegel, 2001, 2008, 2015) require a constant interpretation by the neocortex (especially in the left hemisphere) to maintain the consistency and continuity of consciousness and personal narrative (Gazzaniga, 2012). The brain continues to try to attribute meaning to automatic, unconscious and involuntary responses (*responsiveness*) and is modulated by older motivational systems and oriented toward the monitoring of context. Often, the subsequent narration is poor on relational and semantic levels, but effective in returning a sense of events and coherence with internal states. Conscious attempts (*consciousness*) to attribute sense and meaning to the thematic narrative are more evolved and effective if there are sufficiently good relational occasions. These attempts act through interpersonal co-regulation, based on the activation of polyvagal prosocial systems (Porges, 2001), interpersonal motivations (Liotti and Gilbert, 2011), and affective systems sustained by the activity of the right hemisphere (Schore, 2009). However, the greatest narrative effectiveness is expressed through the semantic sharing in both its linguistic (*storytelling*) and expressive extralinguistic forms. The evolving advantages of the shared narrative of Life Themes also expose individuals to higher risks of interpersonal accidents and failures, and thus to top–down semantic traumas with consequent severe dysregulation of IMS activation and arousal.

### Absent/Negative Narration vs. Present/Positive Narration of the Theme

The narrative of one or more LTs may have been excluded from experience due to the total lack of significant interpersonal occasions related to the semantic content expressed by the Life Theme. It may generate a serious narrative deficit and preclude the possibility of constructing an integrated and harmonic self (van der Hart et al., 2006; Lanius et al., 2010). In other cases, it may be inaccessible to memory because it is associated with severely and repeatedly traumatic relational experiences. It may have been removed to reduce access to excessively painful or disorganized mental or somatic states. It can be systematically avoided not to reactivate conscious traumatic memories. It can be easily accessible but negatively characterized so as to create dysregulated internal states or painful conflicts between parts of the self.

Conversely, it can be derived from rich interpersonal experiences, which are often positive and useful for building a wide and coherent self-concept and world-concept at the semantic level. The narrative of the Life Theme cannot be absent and positive (for a logical constraint) but can be present and negative. In this case it is placed in the axis portion with negative polarity.

### Narration with Low Integration vs. Narrative with High Integration of the Theme

A narration with high integration is rich, coherent, extensive, extended in time, and continuous. It involves an articulated use of semantic, episodic and autobiographical memory. It highlights effective inter-hemispherical communication as well as good connections among cognitive, emotional, arousal, and action levels. It is normally associated with an excellent level of reflective function and Theory of Mind and, in a virtuous recursive process, generates and is supported by a good integration between the parts of the self.

The intersections between the three axes generate four different narrative areas within which the different themes can occupy all the positions arising from the different recombination. Therefore, each individual may have one or more themes located in each different area.

High presence and positivity of narration of the theme, high sharing, and high integration describe the area of wellbeing and resources.

Absence or negativity of narration of the theme, absence of integration, and lack of sharing describe the area of semantic trauma.

Absence or negation of the narration of the theme, but with sufficient integration and sharing describe the area of lack or loss of meaning.

**FIGURE 10:**
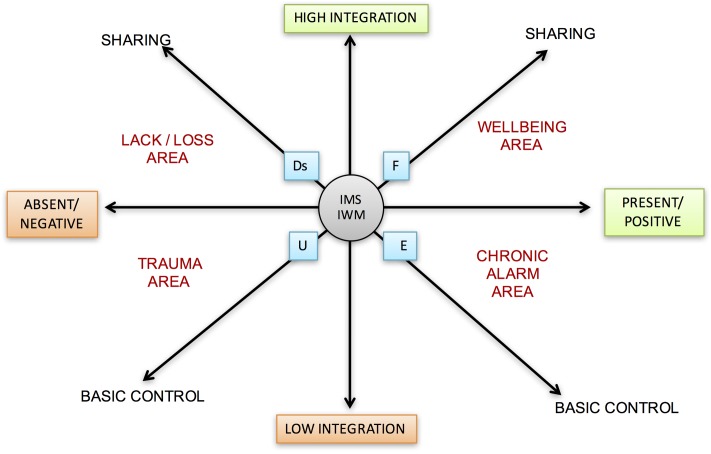
Lines of development of thematic narration. This graphic illustrates the different axes, each of which describes a continuum of possible positions. Within the areas are also placed the connected four states of mind with respect to attachment (F, Ds, E, U). At point 0, attachment representations and the interpersonal motivational systems are represented.

Presence or positivity of the narration of the theme, but poor sharing and integration, describe the area of the chronic semantic alarm. For a graphical representation see **Figure 10**.

## Life Themes, Interpersonal Relationships, and Attachment System

Narrative processes are not only responsible for the harmonization of different selves within the same person, but also of interpersonal integration (Siegel, 2001, 2008, 2015). As social processes, the narration, contents and tone of the story are affected by the influence of those who listen and thus contribute to the building of shared and collective meanings. In the construction of a personal story, attachment stories have a great importance (Bowlby, 1969, 1973, 1980, 1982). These stories are created within the relationship with the parents who, in turn, have a previous story and create a new one with us, laying the foundations for the realization of the goals in a unique way. The first are figures we relate to, the environment they offer us, as well as the cultural context we are part of, which are effective tools to differentiate and create our own personal story (Veglia, 1999).

Some studies (Oppenheim and Waters, 1995; Gini et al., 2007; Vetere and Dallos, 2008) have focused on the link between attachment and narrative based on the narrative assessment that evaluates both the content and structure of stories in childhood. Those studies were guided by the assertion that the particular way of interaction between mother and child is represented in the Internal Working Model of the relationship which will depend, on the one hand, on the flexibility in telling the experiences of attachment and, on the other hand, by the themes and consistency of these tales.

Thus, it was noted that as early as 6 years of age, children with a secure attachment elaborate narratives whose themes reflect the constant presence of parents, accompanied by rich and collaborative interactions and the representation of a good ability to face stressful situations (Oppenheim and Waters, 1995). Moreover, we can find a realistic description of oneself associated with the expression of both positive and negative emotions related to relationships, presented in terms of reflection and negotiation and without excessive anxiety with regard to communication processes (Farrar et al., 1997; Vetere and Dallos, 2008). Story processing by children classified as “insecure” is characterized by the disruption of relationship descriptions, inconsistent responses, or a reduced reflection on resolution strategies for the interpersonal problems. Other studies (Fivush et al., 2006; Fivush, 2008; Haden et al., 2009) identified different forms of mother–child conversation supported by many different elaborative styles with which mothers involve children of pre-school age in dialogs related to the past. The quality of attachment, therefore, can be a predisposing factor in the emergence of psychological disorders if we consider the various styles as meaning dimensions that can be elaborated on different levels of flexibility and generativity (Lambruschi et al., 2004). In disorganized attachment and consequent traumatic development (Liotti and Farina, 2011), self-depiction and autobiographical reconstruction of a person’s experience of painful and fearful situations from which he/she felt overwhelmed may be fragmented and compromised (van der Hart et al., 2006; Dutra et al., 2009).

## The Meaning of LTs in Clinical Psychology

In recent decades, the literature agrees that a narrative approach is a useful clinical tool. In fact, a narrative approach based on the life stories of patients has become the focus of clinical practice (Veglia, 1999; Singer et al., 2008). This approach to psychotherapy focuses on the patient’s life story as an opportunity to co-construct new meanings and readings of past experiences (White and Epston, 1990; Veglia, 1999, 2013; Goncalves et al., 2000; Angus and McLeod, 2004; Singer et al., 2008). This co-construction process, which also involves the sharing of emotionally relevant personal experiences, becomes the foundation for building a “therapeutic alliance” (Adler and McAdams, 2007). According to the authors who adhere to this approach, in the therapeutic work it is important to identify those repetitive patterns of emotions, representations of oneself and others, as well as positive and negative cognitions within autobiographical stories (Angus et al., 2004; Singer et al., 2008).

The development of LTs is the key to feeling like other individuals but, simultaneously, special beings with a specific identity. The individual has the ability and freedom to develop the themes in an original and particular way, but sometimes he/she is restrained by his/her own story, by life events, and the narrative structure of his/her tale. In therapy, in fact, stories that are carried out rigidly and stereotypically represent mostly an adaptation to dominant thinking. A static narrative, devoid of ideas and originality, imposes on the individual limits and constraints that cause a sense of confusion and suffering (Veglia, 1999, 2013). Thus, the therapist’s work involves a re-reading of the patient’s dominant story to offer him/her new narrative alternatives and the opportunity to continue his/her story around more centralized, self-centered themes.

Love, Value, Power, Freedom, Truth and Justice would, therefore, serve as attractors and semantic organizers by returning, on the basis of the occasions and limits that relationships and events offered to the patient, frames of meaning, identity, belongings and a unique and unrepeatable way of being himself/herself.

Unfortunately, autobiographical memory and personal narratives describe the main sources of mental suffering expressed in all its forms.

**FIGURE 11:**
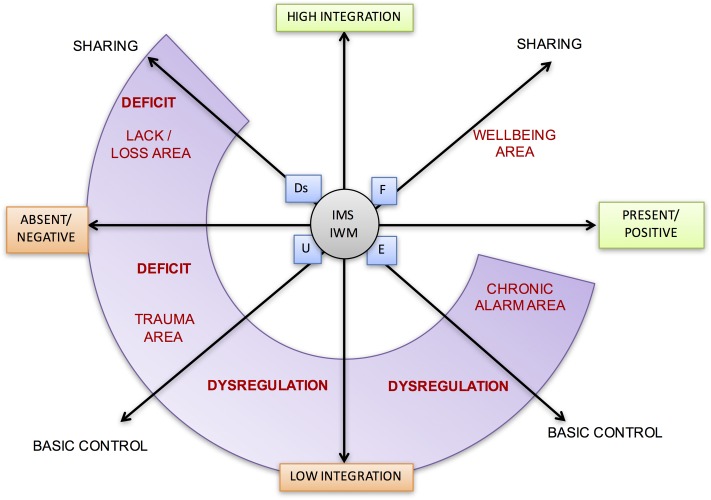
Critical areas in the thematic narration. The schematic shows the deficit or dysregulation areas between the axes.

Using the three axes proposed in this article, namely *presence/absence* of positive or negative narrative occasions, *high/low integration*, and *control/sharing*, it is possible to identify areas of greatest suffering, areas where it appeared (*lack/loss*, *trauma*, *chronic alarm*) and the predominant nature of the deficits and dysregulations of the narrative structure related to the idea of self and the world (see **Figure 11**).

By placing the weighed position of each narrative theme in relation to the three axes in a single map, a configuration of the areas of suffering, available resources and their connections, is useful for narrative understanding (diagnostics) and for the construction of the care plan.

**FIGURE 12:**
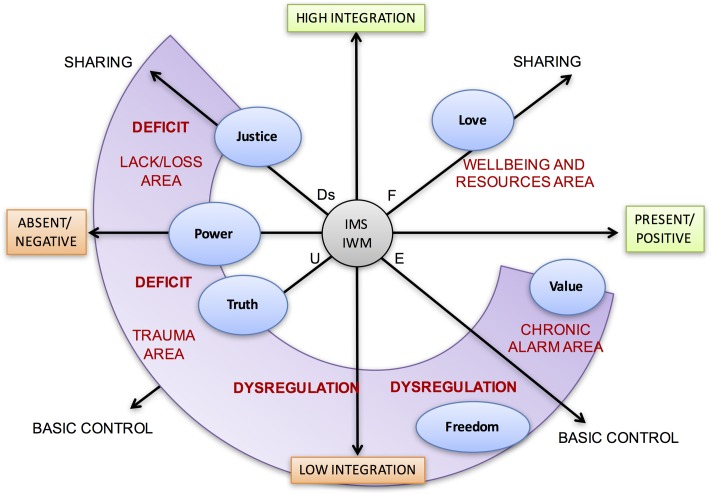
Example of the critical areas in a patient’s thematic narration. The patient suffers from panic attacks with agoraphobia, little sense of self-efficacy, and mild dissociative symptoms. The scheme reassumes the positions of the themes along the axes and within the areas between them.

For example, a graphic representation of the suffering and thematic resources of a patient with a psychiatric diagnosis of panic-attack disorders with agoraphobia, mild dissociative symptoms and severe deficiency of self-efficacy is shown in **Figure 12**.

It is clearly shown that self-representation with others with respect to the Love theme corresponds to a significant resource both in terms of love as well as the ability to give and receive love. Personal value is more uncertain; it suffers from more control, but still lies in an area of wellbeing. Instead, the meaning attributed alternately to Freedom is extremely distressing in terms of dysregulation of narrative control and integration. On the one hand, it is experienced as a strong push to the exploration and rejection of every constraint boundary; on the other hand, it is experienced as irreparable loss if he/she moves away from an attachment figure. This narrative mode is typical of patients with phobic disorders. The theme of personal Power is critical because of a narrative deficit in self-representation within a family narrative and poorly integrated with the strong sense of amiability described previously. From here, the lack of self-efficacy emerges. The traumatic nucleus, which is more difficult to access but is detected by mild dissociative symptoms, is related to the narration of the maternal and inaccessible truth theme, which hides the intriguing dramatic secrets of a patient with great confusion, loss and an overwhelming sense of helplessness.

The final thematic profile shown in **Figure 12** derives, in this case, from the analysis of the therapist’s notes about a therapeutic session. The LT were identified through a coding process (which is already under construction by the Authors) that detects the occurrence of each LT within narrative units through general rules for the analysis. The different axes along which LTs are positioned are therefore considered as Likert scales.

In this case the narrative rereading of the six LTs placed on the three axes and on the consequent map according to the different positions allows to significantly increase the knowledge provided by anamnesis and psychiatric diagnosis, to more structurally and dynamically understand expressive suffering and build a more calibrated and targeted care plan.

In the vast majority of cases, most meaningful content does not emerge from narrative personal stories. However, through conversation, as well as the alternation of active listening to empathic and floating attention, the therapist can approach the most incarnate aspects and even very painful and traumatic contents that cannot be narrated. What matters is attempting to capture the unique way of “making stories” of the patient according to his/her personal interpretation of the reality and in his/her way of telling the cause of his/her suffering. The therapist should, therefore, seek meaningful historical events (i.e., the subjective experience built by selecting meaningful data, arranged in sequence and placed in the flow of subjective time) that are somehow accessible and rebuildable through the memory. Historical consciousness can, therefore, be considered as a tool for re-reading events in another perspective and for building another possible future, because it offers the possibility to rearrange a plot that is very confused and a source of psychic suffering or to rewrite another. Often, whole stories are forgotten, deleted or “normalized” to maintain a coherent narrative plot. In therapy, traces of historical consciousness emerge in the form of discomfort and symptoms; in these cases, the therapist must help the patient to falsify previous apparently probable theories, events, and the world. This strategy provides a second viewpoint to lay the groundwork for negotiating a third meaning and creating a new theory, more coherent with the self, that can orient and support change.

Therefore, the analysis of Life Themes was proposed as a method for entering into the specificity of the patient’s history so that it can be re-read from a new viewpoint (Veglia, 1999, 2013). For example, identifying the various ways in which the patient interprets the theme of love allows the therapist to understand what specific aspect of love generates suffering. In fact, the patient may have problems with his/her lovable-self, loving-self, or loved-self. A person who has never been loved or loved by a “toxic” love may feel loved and contented by the therapist and at each session experience contentment and wonder in seeing him/her again because he/she will have an extra piece of the story to tell (Amanzio and Veglia, 2009). If, instead, the theme of Value becomes a critical topic, the therapist should favor the process of subtracting the critical attributes so that the ontological value of the patient can emerge in an act of self-recognition. Being lovable, with a value and through the power of being oneself, are dynamic and constantly moving conditions that need continuous confirmation from others. The criticality of the Power theme is expressed mainly in the sense of impotence and inability to act, up to the sense of alienation. The Freedom theme also has important clinical implications because each therapeutic act implies the possibility of change and is, therefore, geared to increasing the individual’s degree of freedom.

In the light of these considerations, the work with LTs in psychotherapy allows treatment to go beyond symptomatic resolution to effectively prevent any subsequent side effects because of its ability to affect the patient’s deep existential meaning. The perspective of Veglia (1999, 2013) focuses on this work, which considers clinically the thematic register as one that guides the therapist in collecting the patient’s life story.

## Conclusion and Future Directions

In recent decades, most of the studies focusing on the role of narration in the clinical field decomposed this concept in more descriptive and phenomenological dimensions that can be operationalized. However, what seems to be missing today is a study on the narrative development of specific thematic contents in the clinical field. Little is known about how to empirically translate knowledge on the existence of LTs (Veglia, 1999, 2013), the essential and irreducible organizers of meaning around which the individual builds his/her interpersonal stories and shares them by telling them, for research purposes and the clinical process.

In the cognitive–evolutionary perspective (MacLean, 1984), it is believed that the presented LTs are in continuity with the work of IMS and their representations (Liotti, 2001). In the light of these considerations, a research group within the Department of Psychology at the University of Turin is focusing on systematization of a theoretical model that operationally defines thematic constructs within clinical narratives. The starting point of this empirical study in this sense was the attachment system, that is, the system that primarily contributes to determining self-referential meanings and generating a specific sense of self (Guidano, 1987; Siegel, 2001). The adult narratives of past attachment experiences are the main vehicle for investigating not only the narrative coherence processes, but also structured thematic contents in reference to internal working models. The nature of LTs is supposed to be relational for the basic function of the narration to share meaning; it is equally true that early attachment experiences represent the first interpersonal relationships within which to develop autobiographical narrative processes, autonoetic consciousness and integration mechanisms associated with the attainment of new levels of consistency of the mind (Siegel, 2001). A first step in the exploration of these thematic contents, organized in particular around specific LTs (Veglia, 1999, 2013), took place within the attachment stories (through the use of the adult attachment interview – George et al., 1985, unpublished) to cast a bridge of conjunction between the study of the narrative coherence and semantic investigation. The literature on which LTs hypothesis is based is very broad and heterogeneous with references to various areas of psychology. This can represent a limit but also a challenge for the future to study the model proposed through the analysis of the roots of narration and its contents.

In the literature, there are several tools for assessment of the thematic contents of memories and narratives. They focus mainly on the analysis of specific dimensions of narrative themes, omitting both the multiple facets of the same theme and the holistic view of the thematic content of a narration that takes place around more semantics. A further development of research, that is already a work in progress, is the construction of a reliable system for identifying and coding LTs in therapeutic-session transcripts or semi-structured clinical interviews. The Authors are working to systematize and standardize a reliable coding manual for LTs’ identification, through the definition of general rules for the analysis of their occurrence in narrative units and their positioning along the axes proposed within the theoretical and clinical hypothesis. The identification of LTs *presence/absence*, the assignment of different degrees of intensity and their location on *high/low integration* and *control/sharing* axes could reveal thematic profiles from narrations, that are useful in understanding the patient’s suffering.

## Author Contributions

FV developed the hypothesis of Life Themes and provided the theoretical insights and clinical foundations. GDF reviewed literature and contributed to the conception and develop of the manuscript. Both authors wrote the manuscript and have made substantial, direct and intellectual contribution to the work, and approved it for publication.

## Conflict of Interest Statement

The authors declare that the research was conducted in the absence of any commercial or financial relationships that could be construed as a potential conflict of interest.
